# AI-driven MRI analysis reveals brain atrophy patterns in benign relapsing-remitting multiple sclerosis

**DOI:** 10.3389/fneur.2025.1570566

**Published:** 2025-05-09

**Authors:** M. Niiranen, P. Bendel, J. Koikkalainen, J. Lötjönen, T. Selander, E. Solje, P. Hartikainen, S. Simula, R. Vanninen, A. M. Portaankorva

**Affiliations:** ^1^Department of Neurology, Neuro Center, Kuopio University Hospital, Kuopio, Finland; ^2^Department of Neurology, Institute of Clinical Medicine, University of Eastern Finland, Kuopio, Finland; ^3^Department of Radiology, Institute of Clinical Medicine, University of Eastern Finland, Kuopio, Finland; ^4^Department of Radiology, Imaging Center, Kuopio University Hospital, Kuopio, Finland; ^5^Combinostics Ltd., Tampere, Finland; ^6^Science Service Center, Kuopio University Hospital, Kuopio, Finland; ^7^Department of Neurology, Mikkeli Central Hospital, Mikkeli, Finland; ^8^Clinical Neurosciences, University of Helsinki, Helsinki, Finland

**Keywords:** corpus callosum index, brain atrophy, MRI, benign multiple sclerosis, brain volumetry

## Abstract

**Background:**

The existence and definition of benign multiple sclerosis (MS) remain controversial, particularly given the discrepancy between clinical presentation and underlying imaging changes. In this study, we aimed to investigate the brain atrophy patterns related to benign relapsing-remitting MS (BRRMS), particularly regarding location and extent.

**Methods:**

We analyzed global and regional gray matter (GM) and white matter (WM) volumes, WM lesion load, corpus callosum index (CCI) and corpus callosum area (CCA) in well-defined benign relapsing-remitting MS patients (BRRMS, *n* = 35) compared to healthy controls (HC, *n* = 35). Imaging data were analyzed using an AI-based volumetric analysis MRI (cNeuro^®^) and confirmed visually by an experienced neuroradiologist, ensuring robust validation.

**Results:**

Total brain tissue volume was significantly smaller in patients with BRRMS compared to HC (*p* < 0.001), but the cortical (*p* = 0.011) and cerebral (*p* = 0.002) GM volumes, as well as cingulate gyrus (p=0.032) and entorhinal area volumes (*p* < 0.001), were larger in BRRMS. GM volumes in the postcentral gyrus (*p* = 0.001), precentral gyrus (*p* < 0.001), the medial segment of the precentral gyrus (*p* < 0.001), supplementary motor cortex (*p* < 0.001) and thalamus (*p* < 0.001) were reduced in BRRMS compared to HC. Furthermore, both CCI and CCA were significantly smaller in BRRMS (*p* < 0.001 and *p* = 0.001, respectively).

**Conclusions:**

Despite the overall reduced brain volume compared to HC, distinct cortical regions, especially within the limbic system (i.e., cingulate gyrus and entorhinal area) GM may be relatively well preserved, indicating a possible compensatory volume increase. Based on this study, the corpus callosum is a crucial structure in monitoring disease progression in BRRMS.

## 1 Introduction

Degenerative neuroaxonal loss is considered the primary cause of irreversible physical and cognitive disability in multiple sclerosis (MS) (1). Disease activity varies significantly between patients, with the majority of untreated patients suffering from active relapsing-remitting course. However, a subset of MS patients show minimal disability even decades after symptom onset, forming an entity of so-called benign MS, which has been a subject of debate since the 1950s (2). Currently, no clinical prognostic markers predict the clinical course of MS.

Numerous studies have confirmed that MS patients exhibit an accelerated rate of brain atrophy compared to healthy subjects (3–5). While brain atrophy occurs in normal aging at the rate of 0.1–0.3 % per year, in MS, the annual rate increases to 0.5–1.3 % at all stages of the disease (3, 4). Furthermore, pronounced gray matter (GM) atrophy turns up already at the early stages of relapse-onset MS (6, 7) and primary-progressive MS (PPMS) (8). Brain volume loss has also been identified in benign MS patients presenting only minor clinical disease activity or disability (defined as EDSS equal or < 3.0 and disease duration at least 15 years) (9). The reduction in brain volume in benign MS can be comparable to that seen in secondary-progressive MS (SPMS) when the disease course is prolonged (9). Previous studies have reported a reduction of thalamic (10) and GM volumes in subcortical and frontoparietal regions (11) in benign MS compared to healthy controls (HC).

Several technical methods exist to quantify gray and white matter loss (12). Visual rating scales for assessing global brain atrophy are relatively coarse and prone to rater-related errors, and the method is time-consuming. Automated quantification tools that provide the whole brain and segmental volumetry, as well as lesion volumetry, may aid in evaluating the prognosis and activity of MS and monitoring response to the treatment.

Current automated MRI analysis methods are powerful tools for identifying minimal brain atrophy. However, only a few studies report brain atrophy in mild and benign MS. Our aim was to evaluate the extent and distribution of brain volume loss in benign MS using an automated MRI quantification tool (cNeuro^®^). Easily accessible and usable tools are needed to monitor the treatment response in clinical practice.

## 2 Materials and methods

### 2.1 Patients

The study population consists of patients with benign relapsing-remitting MS (BRRMS, *n* = 35) from the Neurology Outpatient Clinic of Kuopio University Hospital (KUH). Patients were diagnosed with clinically definite MS according to Poser (13) or McDonald criteria (14, 15). Different diagnostic criteria were utilized, as the MS diagnostic criteria have evolved over the years. The same patient population has been included in our previous publication with cNeuro^®^ MRI quantification tool comparing brain volumetry in two different clinical phenotypes of MS (16).

A patient was classified to have BRRMS when the Expanded Disability Status Scale (EDSS) (17) score was ≤ 3 and the disease duration was ≥10 years (18). These patients had never used any disease modifying treatments (DMT) or were stable with the first-line DMT (dimethyl fumarate, glatiramer acetate, interferon or teriflunomide).

Demographic details and MS disease history were retrospectively collected from the patient records (Table 1). Clinical evaluation, including EDSS, was performed by an experienced neurologist at the time of MRI scanning. Disease duration was defined as the time elapsed from the onset symptoms of MS until the MRI scanning. All patients were clinically stable within 1 month before MRI scanning (neither clinical relapses nor cortisone treatments) and with no Gd-enhancing lesions in MRI.

**Table 1:** Demographics of the BRRMS patients.

**Parameters**	**Any DMT *n =* 23**	**No DMT *n =* 12**	***p***
Gender, female *n* (%)	17 (73.9)	11 (91.7)	0.213
Age at the onset symptoms, years (range)	32.2 (14–51)	33.9 (22–46)	0.572
Age at the time of MRI, years (range)	49.1 (32–70)	54.6 (26–66)	0.172
Duration of disease at the time of MRI years (range)	16.9 (12–29)	20.7 (13–33)	**0.031**
Number of relapses at the time of MRI (median, range)	4 (2–10)	3 (1–5)	0.079
EDSS score at the time of MRI (median, range)	2.0 (0–3.0)	1.75 (1.0–3.0)	0.905
**Onset symptoms of MS**, ***n*** **(%)**			
Optic neuritis	6 (26.1)	2 (16.7)	0.529
Sensory symptoms	5 (21.7)	5 (41.7)	0.215
Motor paresis	4 (17.4)	1 (8.3)	0.467
Cerebellar/brainstem symptoms	6 (26.1)	3 (25.0)	0.944
Myelitis	6 (26.1)	5 (41.7)	0.346
**DMT at the time of MRI scanning**, ***n*** **(%)**			
None	2 (8.7)	12 (100)	
Interferone or glatiramer acetate	18 (78.3)	0	
Teriflunomide or dimethyl fumarate	3 (13)	0	

BRRMS, benign relapsing-remitting multiple sclerosis; DMT, disease-modifying treatment; EDSS, Expanded Disability Status Scale; MRI, magnetic resonance imaging. Bold values indicate statistically significant results (*p* < 0.05).

As a control group, we collected age and gender-matched HC (*n* = 35) from an internet-based Open Access Series of Imaging Studies (OASIS; https://www.oasis-brains.org database).

The independent regional ethics committee of Northern Savonia Hospital district, approved this study (44/2014).

### 2.2 MRI acquisition and analysis

The MS patients were referred to MRI with clinical indications. Five different MRI scanner models (1.5- or 3-Tesla) were used. In MS patients, 20% (*n* = 7) of MRI images were scanned with a 3-Tesla scanner. The imaging protocol included a 3-dimensional T1-weighted gradient-echo sequence (3D T1-w) and a fast fluid-attenuated inversion recovery (FLAIR) sequence. The voxel size varied between 0.4 – 1.6 × 0.4 – 1.6 × 0.5 – 2.2 mm in T1 images and 0.4 – 1.3 × 0.4 – 1.3 × 0.6 – 7.0 mm in FLAIR images. Altogether 40% of the 3D T1-w images in MS patients appropriate for volumetric analysis were scanned with Gadolinium (Gd) enhancement. The latest MRI examination, including 3D T1-w images, was chosen to obtain the longest period possible counted from the onset of symptoms.

In HC, OASIS-1 cross-sectional data Siemens Vision 1.5 Tesla brain MRI scanners were used. The imaging protocol used included an MP-RAGE T1-weighed sequence and the voxel size was 1.0 × 1.0 × 1.0 mm.

**Figure 1:**
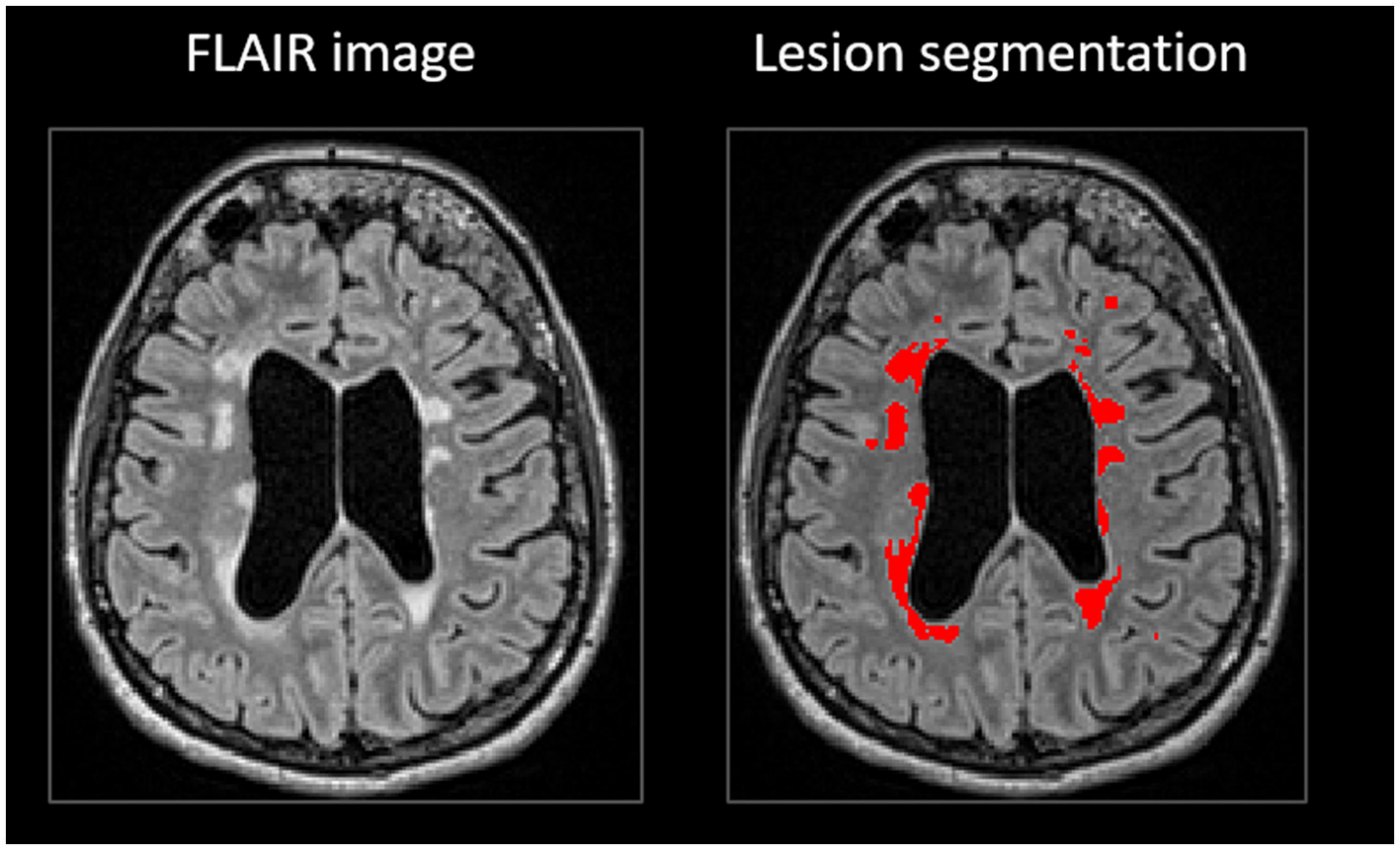
White matter lesion segmentation in cNeuro^®^.

A set of 328 different volumetry and voxel-based morphometry imaging biomarkers was extracted from T1-weighted and FLAIR images using the cNeuro^®^ MRI quantification tool (Combinostics Oy, Tampere, Finland) (19). Images were segmented into 133 brain regions using the multi-atlas segmentation method (102 cortical and 31 sub-cortical regions) (19–21). In this study, results for 33 imaging biomarkers are reported. The WM lesions were segmented as previously described (21, 22), and the lesion volume was reported globally and regionally for the following brain regions: periventricular, subcortical, deep white matter, pons and cerebellum (Figure 1). The method uses the state-of-the-art lesion-filling technique, which removes lesions from images before T1 segmentation. All the quantified variables were normalized regarding age, gender, and head size (23, 24).

**Figure 2:**
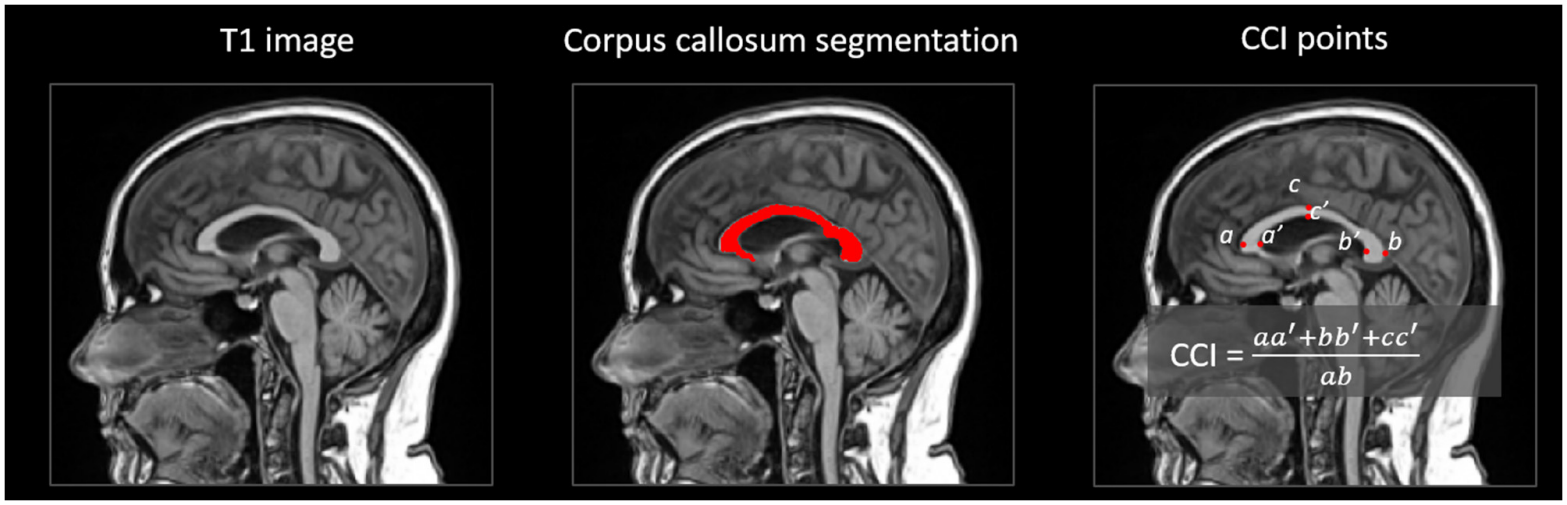
Computation of corpus callosum index (CCI) in cNeuro^®^.

The extraction of the CCI and CCA was not available in cNeuro^®^. Figure 2 visualizes the computation of CCA and CCI. The WM was segmented from the T1-weighted image using the cNeuro^®^ MRI quantification tool. This segmentation was transformed into a template space using affine transformation. The template consisted of an anatomical mean image and a manually drawn mask of CC. The template was non-rigidly registered with the patient image, and the manual CC mask was propagated to the patient image. The CC mask was further dilated, and the CC segmentation of the patient image was obtained by applying the dilated mask to the WM segmentation.

The CCI (25, 26) is based on the distances between six landmarks of CC. These landmarks were automatically detected from the CC segmentation:

a: the most anterior point of CC.
b: the most posterior point of CC.
c: the point with maximal distance from the line between a and b.
a', b', c': the points from the opposite border of the CC.

Seven adjacent slices were analyzed independently to increase the robustness of the automatic analysis. The final CCI was defined by computing the median values for the coordinates of the six landmarks and then computing the CCI using the equation (Figure 2).

The CCA was computed as the mean of the areas of CC segmentation in the seven slices.

As affine registration was used to normalize the template space, the size differences in the CCA between patients were normalized. The CCI is a normalized measure as such.

An experienced neuroradiologist (PB) evaluated the MS patients' MRI images for visual atrophy rating (scale: none, mild, moderate, or strong atrophy) and T2 lesion load rating (scale: lesion amount 0–9, 10–20, 21–40, or over 40 lesions) regarding supratentorial, infratentorial and cortical areas. CCI was determined on a picture achieving and communication system (PACS) workstation on best mid-sagittal T1 weighted images with an established linear measurement technique described earlier (26, 27).

### 2.3 Statistical analysis

Statistical analyses were performed with IBM SPSS Statistics for Windows version 27 (IBM Corp, Armonk, NY). Baseline demographics were expressed as means or medians with ranges or frequencies with percentages. Demographics were compared by *t*-test, Mann-Whitney U test or Chi-square test. Volumetry parameters were expressed as means with standard deviations. Group differences in volumetry parameters were compared by analysis of covariance (ANCOVA) model with adjusting variables (Gd enhancement, and in BRRMS subgroup analysis, also the duration of the disease). Assumption of normality were checked by visually plotting histograms. Volumetry parameters were normally distributed. Results of the ANCOVA model were reported as adjusted mean differences with 95% confidence intervals. *P*-values < 0.05 were set to indicate statistically significant results.

## 3 Results

### 3.1 Clinical characteristics

In both BRRMS and HC groups, women comprised 80% (*n* = 28) of the participants (Table 1.) The mean age at the time of MRI scanning s was 51 years (range 32–70) in both groups. Altogether 32% of BRRMS patients had not received any DMT from the onset of symptoms until the time of MRI scanning. There was no significant difference in age between patients who had received DMT and those who had not. However, disease duration was longer in patients without DMT; thus, it was included as a covariate in subsequent analyses. EDSS levels or the number of relapses did not differ between BRRMS subgroups.

### 3.2 Whole brain volume, GM volumes and lobar volumes in BRMS and HC

Total brain tissue (cerebral GM and WM) volume was reduced in patients with BRRMS (*p* < 0.001) (Table 2). Cerebrospinal fluid (*p* < 0.001) and lateral ventricle volumes (*p* < 0.001) were larger in BRRMS compared to HC. Frontal lobe (*p* = 0.004) and occipital lobe (*p* = 0.020) volumes were larger in BRRMS than in HC.

**Table 2:** Volumetry in patients with BRRMS and healthy controls.

**Variable**	**BRRMS**	**HC**	**Difference (95% CI)**	**p**
***n***	35	35		
**Volumes, ml (mean, SD)**				
Brain tissue (total)	904.37 (50.2)	919.47 (23.04)	−37.87 (−56.48; −19.25)	**< 0.001**
Cortical GM (total)	494.53 (30.21)	485.86 (19.21)	−12.54 (−22.05; −3.03)	**0.011**
Cerebral GM	530.51 (31.91)	525.88 (19.76)	−16.99 (−27.28; −6.69)	**0.002**
Cerebral WM (total)	373.68 (31.81)	394.78 (15.46)	−22.77 (−36.63; −8.91)	**0.002**
Cerebrospinal fluid (total)	56.28 (27.73)	36.76 (11.93)	25.07 (13.53;36.60)	**< 0.001**
Lateral ventricles	48.9 (24.69)	32.42 (10.35)	21.49 (11.27;31.71)	**< 0.001**
**Lobar volumes, ml (mean; SD)**				
Frontal lobes	194.05 (14.52)	192.69 (10.26)	−7.87 (−13.20; −2.54)	**0.004**
Temporal lobes	119.23 (6.89)	115.97 (6.72)	−0.68 (−3.94;2.57)	0.678
Parietal lobes	108.29 (8)	104.64 (5.33)	−0.29 (−3.54;2.96)	0.859
Occipital lobes	73.35 (7.36)	73.05 (6.23)	−3.84 (−7.04;−0.64)	**0.020**
**Regional volumes, ml (mean, SD)**				
Postcentral gyrus	17.86 (1.89)	18.47 (1.5)	−1.49 (−2.33;−0.65)	**0.001**
Post central gyrus (medial segment)	1.18 (0.32)	1.39 (0.36)	−0.16 (−0.35;0.03)	0.089
Precentral gyrus	22.74 (2.78)	23.93 (2.33)	−2.71 (−3.92; −1.50)	**< 0.001**
Precentral gyrus (medial segment)	4.61 (0.71)	5.09 (0.56)	−0.71 (−1.05;−0.38)	**< 0.001**
Supplementary motor cortex	9.01 (1.41)	9.64 (1.05)	−1.30 (−1.91;−0.70)	**< 0.001**
Calcarine cortex	6.44 (1.5)	6.93 (1.78)	−1.09 (−1.95;−0.22)	**0.015**
Medial temporal lobes	18.54 (1.83)	17.89 (1.28)	0.64 (−0.24;1.51)	0.150
Hippocampus	6.54 (0.92)	6.65 (0.65)	−0.20 (−0.63;0.24)	0.377
Thalamus	13.12 (1.81)	14.29 (0.66)	−1.77 (−2.46;−1.07)	**< 0.001**
Anterior cingulate gyrus	8.39 (1.34)	8.04 (1.06)	0.02 (−0.63;0.67)	0.953
Middle cingulate gyrus	9.55 (1.43)	8.55 (0.85)	0.68 (0.04;1.31)	**0.037**
Posterior cingulate gyrus	9.37 (1.17)	8.21 (0.64)	0.75 (0.26;1.23)	**0.003**
Cingulate gyrus (total)	27.31 (2.93)	24.80 (2.09)	1.45 (0.13; 2.76)	**0.032**
Entorhinal area	4.46 (0.54)	3.96 (0.31)	0.47 (0.23;0.71)	**< 0.001**
CCI	0.31 (0.06)	0.37 (0.03)	−0.06 (−0.09;−0.04)	**< 0.001**
CCA, mm^2^	608.22 (116.51)	678.26 (87.61)	−93.21 (−149.25;−37.17)	**0.001**

BRRMS, benign multiple sclerosis; CCI, corpus callosum index; CCA, corpus callosum area; HC, healthy controls; GM, gray matter; MRI, magnetic resonance imaging; n, number; WM, white matter.

p = p-value for group difference, adjusted with Gadolinium-enhancement. Bold values indicate statistically significant results (*p* < 0.05).

The cortical (*p* = 0.011) and cerebral (*p* = 0.002) GM volumes were larger in BRRMS compared to HC. There was no correlation between the WM lesion volumes and cortical GM volumes in BRRMS.

### 3.3 Volumes of WM, CCI, and CCA in BRRMS and HC

Total brain tissue WM volume was reduced in BRRMS compared to HC (*p* = 0.002) (Table 2).

**Figure 3:**
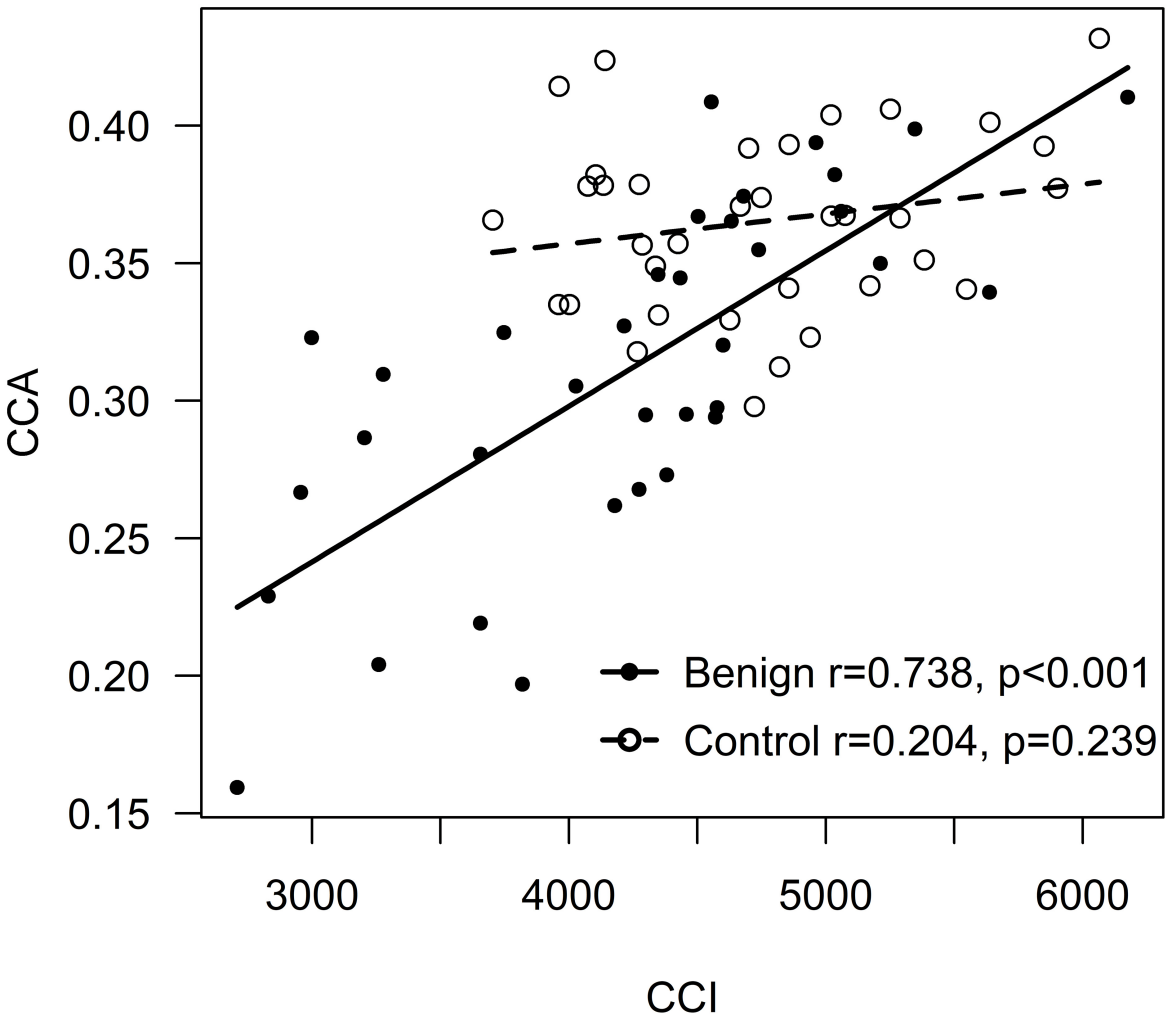
Correlation between CCI and CCA.

Both CCI and CCA were smaller in BRRMS (*p* < 0.001 and *p* = 0.001, respectively) compared to HC. There was a positive correlation between CCI and CCA in BRRMS (*r* = 0.738, *p* < 0.001) but not in HC (Figure 3). A positive correlation was found between CCI and total brain tissue volume in BRRMS (*r* = 0.543, *p* < 0.001) but not in HC, as well as between total brain tissue volume and CCA (*r* = 0.532, *p* = 0.001 and *r* = −0.007, *p* = 0.966, respectively). A negative correlation was found in BRRMS between WM lesion volumes and CCI and CCA (*r* = −0.587, *p* < 0.001 and *r* = −0.663, *p* < 0.001, respectively).

### 3.4 Regional GM volumes in BRMS and HC

Regional GM volumes in the postcentral gyrus (*p* = 0.001), precentral gyrus (*p* < 0.001), the medial segment of the precentral gyrus (*p* < 0.001) and supplementary motor cortex (*p* < 0.001), as well as thalamus (*p* < 0.001) were reduced in BRRMS compared to HC (Table 2). Cingulate gyrus (*p* = 0.032) and entorhinal (*p* < 0.001) volumes were larger in BRRMS compared to HC.

### 3.5 MRI volumetrics in relation to DMT use in BRRMS

**Figure 4:**
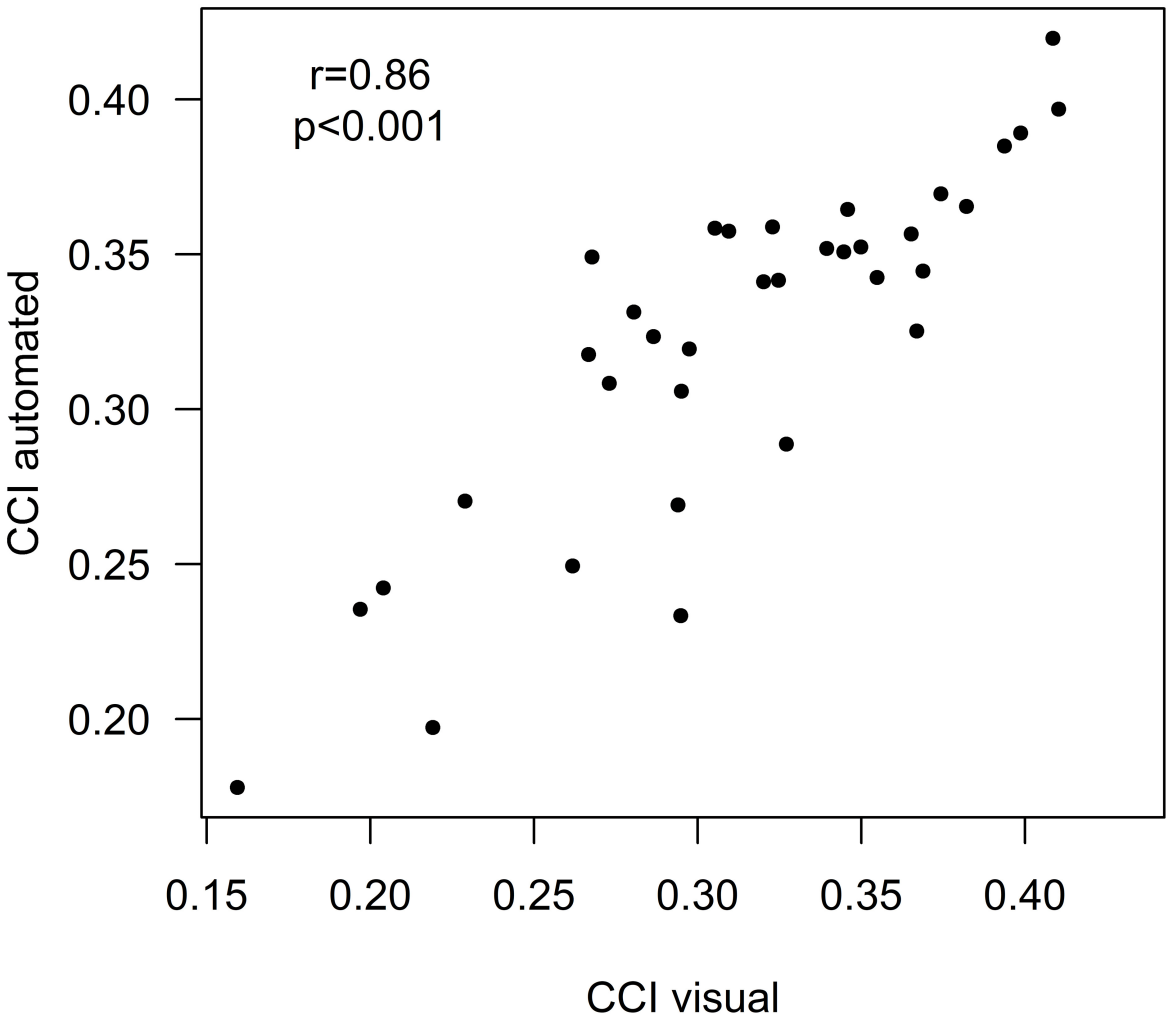
Correlation between visual and automated CCI.

There were no differences in whole brain volumes, cortical total or regional GM volumes, WM volumes or brain lobar volumes between the BRRMS patients with or without DMT (Table 3). CCI and CCA were slightly smaller in patients without a history of DMT, but the results did not differ significantly between these two patient groups. The total, periventricular, juxtacortical and deep WM lesion volumes were larger in patients without a history of DMT compared to those who had used DMT (Table 3). There were no differences in total WM lesion numbers or atrophy rates between the treated and non-treated MS patients in visual assessment. Visual and automated cNeuro^®^ CCI measures were strongly correlated (*r* = 0.86, *p* < 0.001) (Figure 4).

**Table 3:** Volumetry in BRRMS, with treatment and no treatment.

**variable**	**without DMT**	**with DMT**	**Difference (95% CI)**	***p***
*n*	12	23		
**Volumes, ml (mean, SD)**				
Brain tissue (total)	882.93 (48.43)	915.56 (48.37)	19.31 (−15.37;53.99)	0.265
Cortical GM (total)	483.98 (21.28)	500.04 (33.03)	−0.54 (−12.81;11.73)	0.930
Cerebral GM	518.54 (23.39)	536.75 (34.37)	2.14 (−12.20;16.48)	0.763
Cerebral WM (total)	364.36 (32.77)	378.54 (30.89)	16.39 (−10.18;42.96)	0.218
Cerebrospinal fluid (total)	65.97 (34.29)	51.22 (22.85)	−13.62 (−36.09;8.85)	0.226
Lateral ventricles	58.19 (30.87)	44.06 (19.84)	−13.42 (−33.30;6.46)	0.178
**Visual atrophy rating (%)**				0.077
No atrophy	6 (50)	15 (65.2)		
Mild atrophy	1 (14.3)	6 (26.1)		
Moderate atrophy	3 (25)	2 (8.7)		
Strong atrophy	2 (16.7)	0		
**Lobar volumes, ml (mean; SD)**				
Frontal lobe	189.44 (10.25)	196.46 (15.99)	1.30 (−5.74;8.34)	0.708
Temporal lobe	117.86 (7.09)	119.94 (6.84)	−1.31 (−5.41;2.80)	0.522
Parietal lobe	106.02 (7.18)	109.48 (8.3)	0.22 (−5.21;5.64)	0.935
Occipital lobe	70.99 (4.78)	74.58 (8.22)	−0.63 (−5.11;3.85)	0.778
**Regional volumes, ml (mean; SD)**				
Postcentral gyrus	17.58 (1.54)	18.01 (2.07)	−0.45 (−1.75;0.86)	0.492
Postcentral gyrus (medial segment)	1.2 (0.25)	1.17 (0.36)	0.00 (−0.27;0.27)	0.991
Precentral gyrus	22.21 (1.88)	23.02 (3.15)	−0.15 (−1.80;1.49)	0.850
Precentral gyrus (medial segment)	4.73 (0.55)	4.54 (0.78)	−0.37 (−0.89;0.15)	0.154
Supplementary motor cortex	8.53 (1.03)	9.26 (1.53)	0.29 (−0.67;1.25)	0.542
Calcarine cortex	6.38 (1.49)	6.46 (1.54)	−0.61 (−1.71;0.49)	0.265
Medial temporal lobes	17.81 (1.16)	18.92 (2.01)	1.19 (−0.30;2.68)	0.115
Hippocampus	6.25 (0.63)	6.69 (1.01)	0.52 (−0.24;1.28)	0.170
Thalamus	12.48 (2.28)	13.46 (1.45)	0.57 (−0.83;1.97)	0.410
Anterior cingulate gyrus	8.09 (1.38)	8.54 (1.33)	0.30 (−0.79;1.39)	0.577
Middle cingulate gyrus	9.85 (1.13)	9.4 (1.57)	−0.73 (−1.85;0.40)	0.197
Posterior cingulate gyrus	9.21 (1.04)	9.46 (1.24)	−0.04 (−0.94;0.85)	0.921
Entorhinal area	4.2 (0.4)	4.59 (0.56)	0.33 (−0.09;0.75)	0.119
CCI	0.28 (0.07)	0.33 (0.05)	0.04 (−0.01;0.09)	0.143
CCA, mm^2^	588.42 (119.39)	618.55 (116.07)	12.68 (−83.98;109.34)	0.791
CCI, visual analysis	0.30 (0.06)	0.34 (0.05)	0.03 (−0.01; 0.08)	0.161
**Volumes of WM lesions, ml (mean; SD)**				
Total	23.84 (18.71)	13.25 (6.47)	−12.41 (−22.27;−2.54)	**0.015**
Periventricular	5.37 (5.75)	1.52 (1.45)	−3.97 (−6.92;−1.01)	**0.010**
Juxtacortical	3.23 (3.87)	1.03 (1.14)	−2.46 (−4.41;−0.51)	**0.015**
Deep white matter	12.69 (9.49)	7.88 (4.33)	−5.97 (−11.36;−0.58)	**0.031**
Pons	0.05 (0.07)	0.05 (0.15)	0.00 (−0.10;0.11)	0.972
Cerebellar	0.04 (0.08)	0.02 (0.05)	−0.02 (−0.07;0.03)	0.409
Infratentorial	0.09 (0.11)	0.07 (0.19)	−0.02 (−0.16;0.12)	0.778
**Total lesion count in numbers, visual analysis (%)**				0.224
0–9	2 (16.7)	6 (26.1)		
10–20	0	5 (21.7)		
21–40	4 (33.3)	6 (26.1)		
over 40	6 (50)	6 (26.1)		

BRRMS, benign multiple sclerosis; CCI, corpus callosum index; CCA, corpus callosum area; DMT, disease-modifying treatment; GM, gray matter; MRI, magnetic resonance imaging; n, number; WM, white matter.

p = p-value for group difference, adjusted with the duration of disease and Gadolinium-enhancement. Bold values indicate statistically significant results (*p* < 0.05).

## 4 Discussion

Here we aimed to assess atrophy patterns and the rate of atrophy in BRRMS using an AI-based volumetric assessment tool cNeuro^®^. We found reduced total brain volumes and larger CSF volumes in BRRMS patients compared to HC. However, total cortical and cerebral GM volumes were in BRRMS than in HC, which may indicate yet unknown compensatory mechanisms. Notably, increased GM volumes were identified particularly in the limbic areas (in the entorhinal cortex and cingulate gyrus). To our knowledge, this finding has not been reported before.

There are few previous studies reporting brain volumes in benign MS, with controversial findings. Some studies have reported reduced cortical and deep GM volumes and reduced normalized whole brain volumes in benign MS (9, 11, 28). Total brain volume, and GM volume are known to increase temporarily during relapses due to inflammation-induced edema (29).

Global and GM volume loss is correlated with disability progression in MS (30, 31). The association of GM volume with clinical symptoms or disease progression has also been studied in Alzheimer's disease (AD). It has been shown that some elderly people with AD neuropathology do not develop dementia. These non-demented individuals with AD neuropathology are found to have overall GM volume increased and cingulate gyrus thicker compared to patients with clinically demented AD (32). Possible mechanisms are thought to be higher education and cognitive reserve, but also compensation of neural atrophy and reduced neuroinflammation by decreased glial activation. Similarly, the concept of higher maximal lifetime brain growth (MLBG) has been linked to preserved cognitive and motor functions in MS. The brain reserve hypothesis suggests that people with larger MLBG have a better reserve against cognitive impairment (33). In a 5-year follow-up study, MS patients with larger MLBG were at lower risk for disability progression measured by EDSS change (34).

In the present study, we took several precautions to ensure the accuracy of our findings. Patients with recent relapses and glucocorticoid treatments, as well as patients with Gd-enhancing lesions in MRI were excluded from the analyses. Given the extended period over which data were collected, only 40% of the MRI scans included Gd-enhancement. Nevertheless, Gd enhancement was taken into account as a covariate in the analysis, and our results remained consistent even without this covariate. Therefore, the increased total cortical GM volumes in BRRMS cannot be explained by cortical inflammation.

Our finding of increased GM volumes in BRRMS, especially in the limbic areas, is noteworthy. The limbic system plays a crucial role in behavior, emotions, the reward-pleasure system, and higher cognitive functions (35). Injury to the limbic system has been associated with cognitive dysfunction in MS (36). Previous functional MRI studies have suggested the compensatory cortical mechanisms in benign MS (37). These changes in the limbic system warrant further investigations in larger cohorts of early and mild MS cases.

Interestingly, we did not find a correlation between WM lesion volumes and cortical total GM volumes. Typically, higher WM lesion volumes are associated with lower GM volumes or lower cortical thickness. This has been the most consistently observed in the early stage of relapsing MS but less in PPMS (38, 39). The lack of correlation between WM lesion and cortical total GM volumes in our study may suggest that BRRMS and PPMS share some common characteristics.

As expected, thalamic volumes were reduced in BRRMS. This reduction in benign MS has been reported previously and may represent a typical change occurring already in the early disease course. It is likely characteristic of MS itself and purely reflects the vulnerability of the thalamus to the MS pathology (10, 40).

Atrophy of CC has been associated with the level of disability in MS and to correlate with GM atrophy (26, 41, 42). The CC is particularly affected by focal demyelination and Wallerian degeneration in the pathogenesis of MS (43). On the other hand, CC is resistant to age-related changes in healthy individuals (44, 45), making it a relevant structure for a brain atrophy marker in degenerative diseases. Both the CCI and CCA have been demonstrated as reliable markers of brain atrophy in MS (26, 41, 46). The majority of previous analyses using these markers have been conducted with non-automated methods, which are time-consuming and prone to rater-related errors (26, 41, 47, 48). However, more recent studies have applied automated MRI methods (16, 46). In our study, both CCA and CCI correlated with whole brain volume in MS patients but not in HC. Additionally, we found a negative correlation between WM lesion volumes and CC measurements, similar to previous reports, suggesting that CC atrophy is related to both WM and GM pathology as well as Wallerian degeneration (41).

WM lesion volumes were higher in patients who had never been treated with DMT. This finding suggests that subclinical inflammatory activity may exist even in the seemingly mild and benign MS, supporting the use of DMT in managing the benign course of the disease (49, 50). However, there were no differences in total brain volumes, cortical and deep GM volumes, as well as WM volumes in the treated and non-treated BRRMS patients. Additionally, CCA and CCI were similar between these groups. These results indicate that while DMTs may reduce inflammatory activity, they do not appear to prevent neurodegeneration in BRRMS. Current DMTs have proven effective in reducing WM inflammatory lesions and preventing relapses, but they may not directly impact the neurodegenerative processes that continue in the background. Neurodegeneration in MS is a complex, multifactorial process driven by mechanisms such as chronic demyelination, axonal loss, mitochondrial dysfunction, and microglial activation (51). Thus, patients who benefit a reduction in inflammatory lesions due to DMT treatment, may still experience axonal injury and brain atrophy due to these neurodegenerative processes. Neurodegeneration in MS seems to progress independently of inflammation and remains largely unaffected by current DMTs (49, 50).

The strengths of our study include the detailed clinical characteristization and thorough EDSS evaluation conducted for each patient. Also, the duration of the disease in patients with BRRMS exceeds 10 years, which is a commonly used criterion for benign MS (18). However, the main limitation of our study is the lack of cognitive testing. Some patients with minimal motor disability may still suffer from cognitive decline, depression, and fatigue, are important factors of overall disability (52, 53). The definition of benign MS is a retrospective judgment. Over the long follow-up period (i.e., more than 20 years), most patients initially classified as having benign MS eventually develop cognitive decline and overall disability, even in the absence of clinical relapses and despite mild neurological signs (9, 52, 54, 55). Therefore, a longitudinal multicenter study with a significantly longer timeline, with cognitive testing and assessments of fatigue in correlation with MRI volumetrics, is necessary to better define the characteristics of what could be considered a “truly benign MS”.

Due to the retrospective nature of MRI scannings, the imaging protocols, scanners, and voxel sizes varied among the MS patients. This may have influenced cortical GM measures but not in other volume measures. We acknowledge that the use of different scanners forms a possible bias, but the normalization of the structures and consistency with previous studies using the same algorithm suggest that this bias did not significantly affect our results (19, 21, 56). Moreover, in previous studies with the FreeSurfer structural tool, the use of multiple different MRI scanners and pulse sequences did not significantly affect cortical thickness measurements (57, 58). The test-retest variability in the cNeuro^®^ MRI quantification tool between different scanners is two to three times greater than with a single scanner, which is equal to other methods (59). Furthermore, voxel size variation does not appear to impact the results obtained with the cNeuro^®^ MRI quantification tool. Although the automated cNeuro^®^ tool cannot distinguish between demyelinating and vascular lesions, visual analysis ensures that the lesion characteristics in our study are typical for demyelination. Thus, we consider our results logical and suggest that the methodology used is robust.

## 5 Conclusions

This study provides valuable and novel insights into the brain atrophy patterns associated with BRRMS. Our findings suggest that while total brain volume is significantly reduced in BRRMS patients compared to HC, certain cortical regions, particularly within the limbic system, may exhibit relative preservation or even compensatory volume increases, but the plausible mechanism remains unsolved. The corpus callosum seems to be a relevant marker for even minor atrophy and is easily analyzed with automated quantification. The consistent reduction in CCI and CCA may provide a useful clinical biomarker for monitoring BRRMS.

## Data Availability

The datasets presented in this article are not readily available because the generated datasets are partly under the copyright of Combinostics Ltd. Requests to access the datasets should be directed to juha.koikkalainen@combinostics.com.
